# The Role of Bifidobacterium in COVID-19: A Systematic Review

**DOI:** 10.3390/life13091847

**Published:** 2023-08-31

**Authors:** Clarissa Reginato Taufer, Pabulo Henrique Rampelotto

**Affiliations:** 1 Graduate Program in Genetics and Molecular Biology, Universidade Federal do Rio Grande do Sul, Porto Alegre 91501-970, Brazil; 2 Bioinformatics and Biostatistics Core Facility, Instituto de Ciências Básicas da Saúde, Universidade Federal do Rio Grande do Sul, Porto Alegre 91501-970, Brazil; 3 Graduate Program in Biological Sciences: Pharmacology and Therapeutics, Universidade Federal do Rio Grande do Sul, Porto Alegre 91501-970, Brazil

**Keywords:** SARS-CoV-2, microbiota, microbiome, bacteria, probiotics

## Abstract

The COVID-19 pandemic, caused by the SARS-CoV-2 virus, mainly causes respiratory and intestinal symptoms and changes in the microbiota of patients. We performed a systematic search in major databases using “*Bifidobacterium*” and “COVID-19” or “SARS-CoV-2” as key terms to assess the relationship of the genus to COVID-19. After the selection steps, 25 articles were analyzed. Of these, eighteen were observational, and seven were interventional articles that evaluated the use of *Bifidobacterium* alone or in mix as probiotics for additional treatment of patients with COVID-19. All stages and severities were contemplated, including post-COVID-19 patients. Overall, *Bifidobacterium* was associated with both protective effects and reduced abundance in relation to the disease. The genus has been found to be abundant in some cases and linked to disease severity. The studies evaluating the use of *Bifidobacterium* as probiotics have demonstrated the potential of this genus in reducing symptoms, improving pulmonary function, reducing inflammatory markers, alleviating gastrointestinal symptoms, and even contributing to better control of mortality. In summary, Bifidobacterium may offer protection against COVID-19 through its ability to modulate the immune response, reduce inflammation, compete with pathogenic microbes, and maintain gut barrier function. The findings provide valuable insights into the relationship between the disease and the genus *Bifidobacterium*, highlighting the potential of microbiota modulation in the treatment of COVID-19.

## 1. Introduction

The COVID-19 pandemic has had a significant impact on the world, causing widespread illness, death, and economic disruption. The pandemic is caused by the SARS-CoV-2 virus, which primarily affects the respiratory system. While much research has focused on developing effective treatments and vaccines for COVID-19, there is also increasing interest in understanding the underlying mechanisms of how SARS-CoV-2 infection changes human physiology and metabolism. Several studies have shown that the human microbiome plays a crucial role in modulating the immune system and may therefore influence the severity of COVID-19 [1]. One bacteria genus that has gained attention in this regard is *Bifidobacterium*.

*Bifidobacterium* is a genus of Gram-positive, anaerobic bacteria that are commonly found in the human gut microbiome. These bacteria are known for their immunomodulatory properties and have been shown to have a beneficial impact on human health [2]. The abundance of *Bifidobacterium* in the gut has been linked to a reduced risk of various diseases, including gastrointestinal disorders, metabolic disorders, and respiratory infections. Given the role of the gut microbiome in regulating the immune response, there is interest in exploring the potential role of *Bifidobacterium* in COVID-19.

In this work, we explored the relationship between the genus *Bifidobacterium* and COVID-19, highlighting the ecological as well as genetic and functional features of bifidobacteria residing in the gut and in the upper respiratory tract. Through a systematic review of the existing literature, our aim was to identify and synthesize the findings of studies investigating the role of *Bifidobacterium* in regulating the immune response to SARS-CoV-2 infection and the potential impact of bifidobacteria on the severity of COVID-19. The results of this study will provide insight into the potential role of bifidobacteria in COVID-19 and may inform future research on the use of microbiome-based interventions to reduce the severity of the disease.

## 2. Materials and Methods

### 2.1. Search Strategy and Selection Criteria

A systematic literature search was conducted using the PubMed, Embase, Scopus, and Web of Science databases. The search was limited to articles published from January 2020 to May 2023. The search terms used were “*Bifidobacterium*” and “COVID-19” or “SARS-CoV-2”. The strategy was developed using medical subject headings (MeSH) in the PubMed database and adapted for other databases. In addition, the search was narrowed down using filters available in the databases to retrieve works specifically in the English language. The expanded search strategy for each database is presented in Supplementary File S1. Studies that did not meet these criteria were excluded. Review articles, letters, case reports, book chapters, conference abstracts, notes, editorials, in vitro, and animal studies were also excluded. References cited by selected articles were also evaluated and added if they were in accordance with the inclusion criteria.

**Figure 1:**
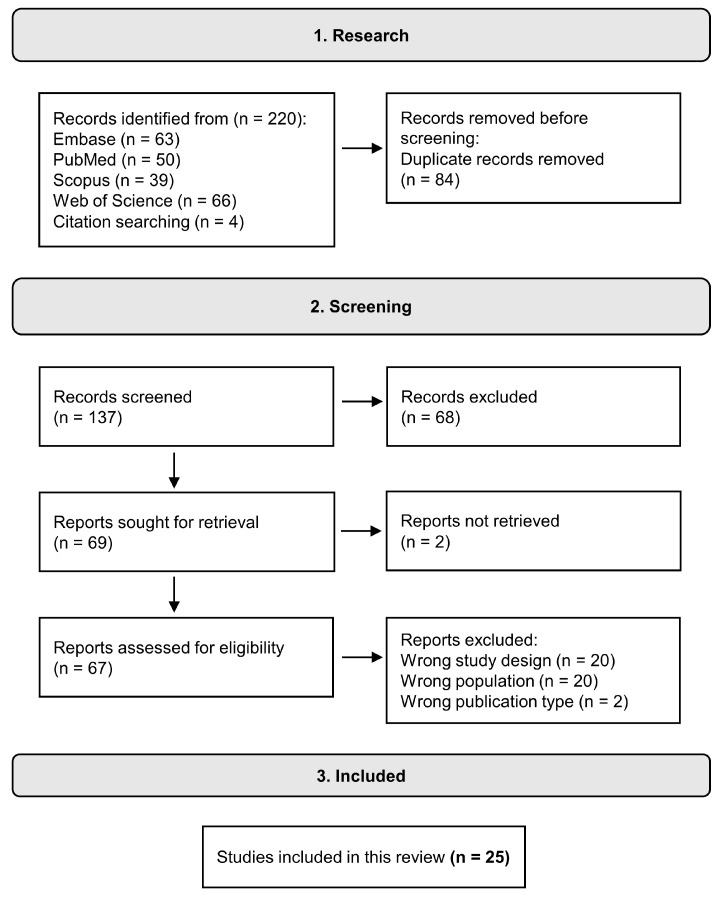
Flow diagram of the database search protocol.

Articles returned from searches in all databases were initially evaluated using the Rayyan web platform to identify and exclude duplicates [3]. Afterward, the works were evaluated by abstract to identify the inclusion and exclusion criteria, or, if necessary, by the full text. Papers that met the inclusion criteria were evaluated using the full text, and data extraction was carried out. Figure 1 presents the flow diagram of the search we performed based on the criteria described above, as well as the number of articles included, and their distribution based on selected criteria.

This systematic review was conducted in accordance with the Preferred Reporting Items for Systematic Reviews criteria and Meta-Analyses (PRISMA) guidelines [4].

### 2.2. Data Extraction and Analysis

Data for each paper were collected in an Excel spreadsheet and consisted of general information about the work (name of the work, authors, journal, DOI, year, country, type of study), information about the study population (number of individuals, severity of disease), methodology (type of sample, NGS technology, type of sequence, reference databank), and results.

For quality assessment, we used the Joanna Briggs Institute’s critical appraisal tools for case–control, cohort, and cross-sectional studies [5]; Cochrane Risk of Bias 2 (RoB2) [6] for randomized intervention studies; and ROBINS-I [7] for non-randomized interventional studies.

## 3. Results

### 3.1. Selected Studies

Table 1 presents the 18 articles that identified changes in the diversity and abundance of the *Bifidobacterium* genus in samples from COVID-19 patients [8,9,10,11,12,13,14,15,16,17,18,19,20,21,22,23,24,25]. The studies were conducted in different countries and involved different patient populations. The country with the highest number of works is China, with six papers, followed by Italy with three and the USA with two. The other countries (Switzerland, Ireland, Brazil, Russia, Turkey, Germany, Iran, and Bangladesh) are represented with only one work. Worthy of note, the work published by the group from Iran, re-analyzed samples deposited in a database from an original Chinese paper. Most of the studies used the 16S rRNA gene amplicon sequencing technique to identify changes in the gut microbiome in adult patients with COVID-19 (stool samples). Only four studies evaluated the oral and/or naso-oropharyngeal microbiota and only two studies evaluated the microbiota of both respiratory systems and the intestinal system. One study evaluated the microbiota of children and adults, and two evaluated children’s microbiota only.

**Figure 2:**
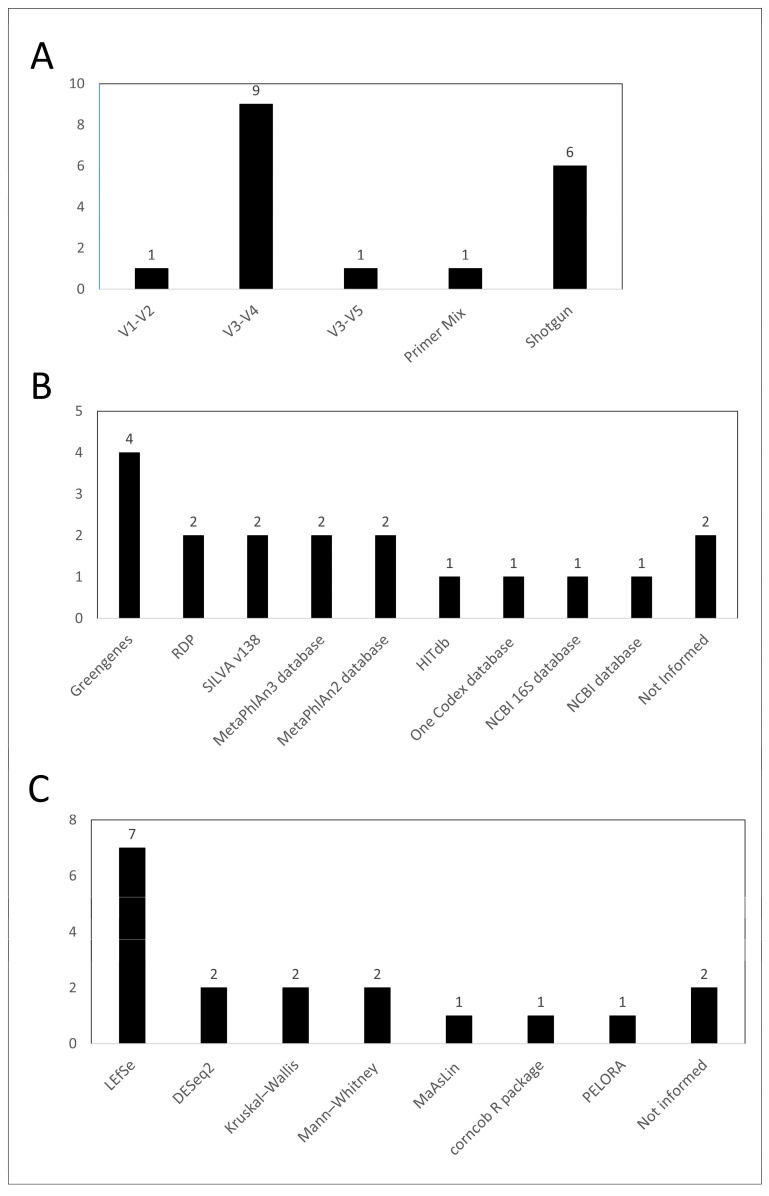
Summary of the microbiome methods used to identify and quantify the *Bifidobacterium* genus. Bar graph for type of sequencing and amplicon regions (**A**), databases used for taxonomy (**B**), and statistical analysis used for assessing the genus abundance variation (**C**).

The V3–V4 region was the most used amplicon sequencing technique (nine works), while the V1–V2 and V3–V5 regions were used in only one work each (Figure 2A). In addition, one work analyzed the microbiome by the set of regions V2-4-8, V3–V6, and V7–V8. The shotgun metagenomic technique was applied in 6 studies. The choice of database used for taxonomy assignment varied significantly among the studies (Figure 2B). In addition, two studies did not specify the database used. Regarding the statistical analysis used to identify if the *Bifidobacterium* genus was differentially abundant between groups, Linear discriminant analysis Effect Size (LEfSe) was the most used analysis method among studies (7/18) while two studies did not clearly mention which method was used (Figure 2C).

Table 2 presents seven studies specifically investigating the protective role of probiotic strains of *Bifidobacterium* in the management of COVID-19 [26,27,28,29,30,31,32]. One study evaluated the use of only one strain of *Bifidobacterium*, the other studies evaluated a mix of probiotics, containing different species of *Bifidobacterium* and other genus. Italy was the country with the most studies (four), and Turkey, China, and Russia presented just one. There are two prospective studies, four retrospective studies, and one post-COVID-19 study. Section 4.3 discusses in detail the results found.

### 3.2. Risk of Bias and Quality Accessment

**Figure 3:**
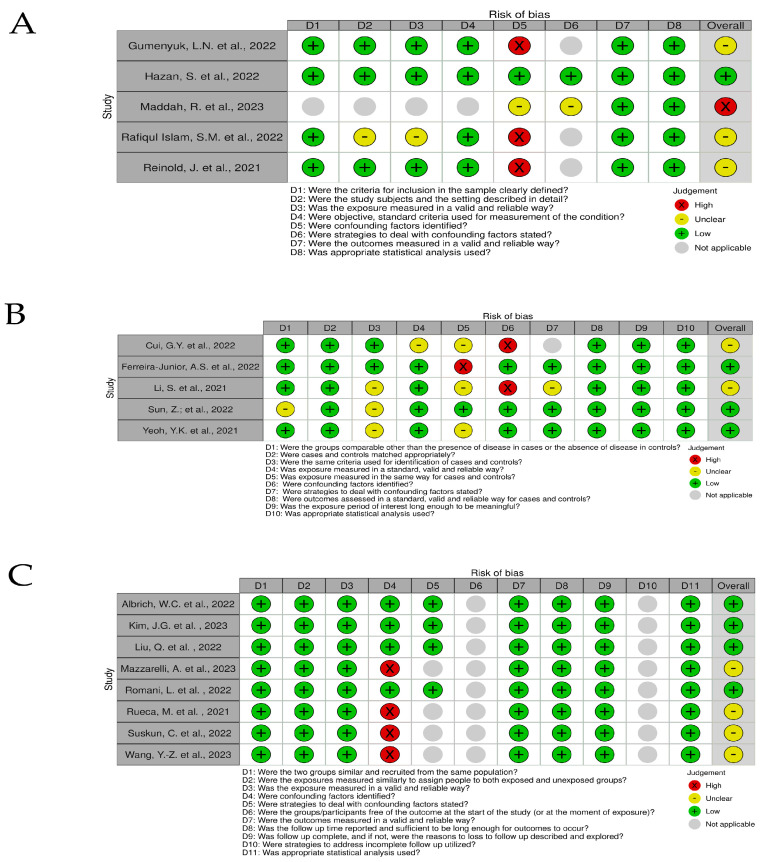
Traffic light chart for risk of bias analysis for cross-sectional studies (**A**) [11,12,16,18,19], case-control studies (**B**) [9,10,14,22,25], and cohort studies (**C**) [8,13,15,17,20,21,23,24].

**Figure 4:**
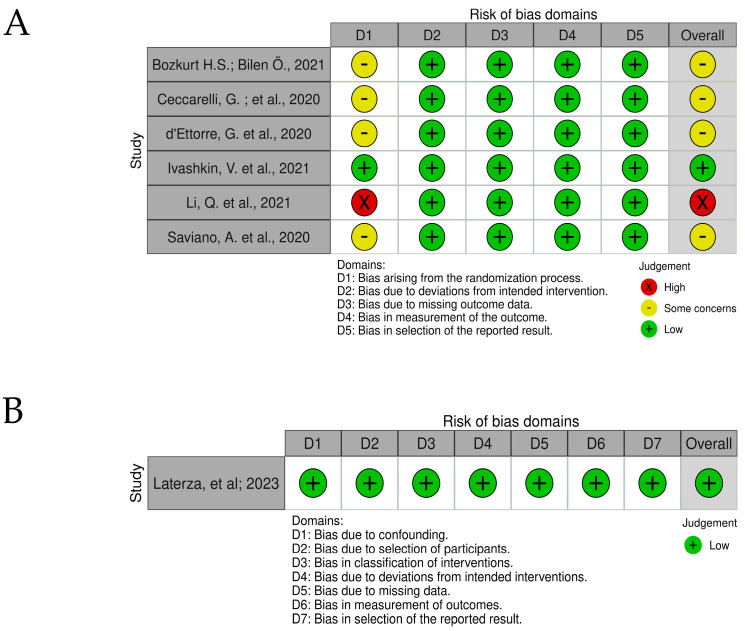
Traffic light chart for risk of bias for randomized intervention studies (**A**) [14,26,27,28,29,32] and non-randomized intervention studies (**B**) [30].

Risks of bias are shown in traffic light graphs in Figure 3 and Figure 4. For cross-sectional and case-control studies, the biggest limitation found was the failure to identify confounding factors. The study carried out by Maddah, and collaborators (2023) [16] was a reanalysis of samples from publicly available COVID-19 patients, so the work does not provide specifications about patients and controls. For cohort studies, the initial assessment of the outcome does not match (D6), which impairs the risk of bias assessment. In randomized intervention studies, Bozkurt; Bilen (2021) [26], Ceccarelli, et al. (2020) [27], and d’Ettorre, et al. (2020) [28] retrospectively evaluated the effects of using probiotics in the treatment of COVID-19 in patients who underwent probiotic supplementation as an alternative part of treatment. Li, et al. (2021) [31], administering probiotics to patients, was defined by the medical team. The work of Ivashkin, et al. (2021) [29] was defined in the text as “per-protocol analysis because there was no final point to perform intention-to-treat analysis”.

Laterza, et al. (2023) [30] study evaluated the administration of probiotics in patients for 8 weeks after hospital discharge (before-and-after). Inclusion and exclusion criteria were well defined in the work and the control samples were from the same patients before the start of the intervention, thus excluding possible risk of bias related to confounding factors, such as underlying diseases.

## 4. Discussion

The results of this systematic review indicate that there is an intrinsic relationship between *Bifidobacterium* and COVID-19. The studies showed that COVID-19 is associated with changes in the gut and nasal microbiome, including a decrease in the abundance of *Bifidobacterium*. The presence of *Bifidobacterium* was associated with a reduced risk of severe COVID-19. However, the mechanisms behind this association are not yet fully understood. The discussion section of this article was structured to fill this gap and to provide a comprehensive overview of the relationship between *Bifidobacterium* and COVID-19.

In Section 4.1, we introduce the *Bifidobacterium* genus, highlighting its importance as a probiotic and its role in maintaining gut health. Section 4.2 presents the evidence linking *Bifidobacterium* to COVID-19, including case-control, observational, and transactional studies. In Section 4.3, we discuss the potential of *Bifidobacterium* as a therapeutic agent for managing COVID-19, including studies that have investigated the potential protective effects of this probiotic against the virus. Section 4.4. presents the risk of bias and limitations of included studies. Section 4.5 reviews the evidence linking *Bifidobacterium* to other viral infections, providing insights into the potential mechanisms of action underlying its antiviral effects. Finally, in Section 4.6, we delve into the mechanisms of protection of the *Bifidobacterium* genus in COVID-19, exploring how this probiotic may modulate the immune response to the virus and contribute to the prevention and management of the disease.

### 4.1. The Bifidobacterium Genus

The *Bifidobacterium* genus, (Actinobacteria phylum, Bifodobacteriaceae family), was first identified in 1900 by Tissier in the feces of breast-fed infants, named *Bacillus bifidus* [33]. It is described as a rod-shaped, non-gas-producing, anaerobic microorganism with bifid morphology, generally characterized as Gram-positive, non-spore producing, non-motile, and catalase-negative anaerobes, with high C+G composition [34]. At first, it was described as a member of the Lactobacteriaceae family by Orla-Jensen, who in 1924 proposed to be a separate species, being confirmed in more detailed studies by Dehenert in 1957 and by Reuter in 1963 [33].

They are found in various ecological niches, but most species are found in the gastrointestinal tract of humans and other mammals, being a common genus of the intestinal microbiota. It has 94 recognized (sub)species and constitutes the deepest-branched lineage of the Actinobacteria phylum [35].

The *Bifidobacterium* genus is widely studied for conferring benefits, mainly to human health. Evidence indicates that bifidobacteria in the intestines of mammals support the development of the immune system, improve homeostasis and intestinal function, promote the integrity of the intestinal barrier, and reduce the appearance of some intestinal diseases, in addition to protecting the host against the proliferation of pathogens [36,37,38]. They are also producers of various metabolites, such as vitamins and short-chain fatty acids (SCFAs), which have a beneficial impact on the host and other intestinal microorganisms [39].

### 4.2. Evidence in COVID-19

In COVID-19, the *Bifidobacterium* genus was identified at lower levels in high-risk adult patients [8], and in pediatric patients compared to controls [20,24], in addition to being negatively associated with the severity of the disease [8,20]. Less severe clinical outcomes were associated with the genus [8].

Similar results were found by Hazan et al. (2022), Reinold et al. (2021), and Liu et al. (2022) where the relative abundance was significantly decreased in COVID-19 patients [12,15,19]. Increased severity was also associated with decreased relative abundance [12]. Linear discriminant effect size analysis (LEfSe), Wang et al. (2023) comparing stool samples, identified the genus *Bifidobacterium* with higher abundance in healthy controls than COVID-19 patients [24], and was identified as a biomarker for SARS-CoV-2 negative patients by Reinold et al. (2021) [19].

Comparisons between patients admitted to the ICU and patients who did not require ICU stay were also performed. The *Bifidobacterium* genus was significantly more abundant among patients who did not require ICU stay (saliva samples) [13]. Furthermore, greater abundance of the genus was associated with lower levels of IL-17F and monocyte chemoattractant protein-1 (MCP-1). Rueca et al. (2021) identified complete genus depletion exclusively in ICU COVID-19 patients [21]. However, in comparisons between moderate and severe groups, *Bifidobacterium* was among the eight genera that were not represented differently between the two groups [17].

At the species level, the *Bifidobacterium pseudocatenulatum* species depletion has been identified in patients with COVID-19 compared with healthy controls [22], a significant decrease in the abundance of *Bifidobacterium adolescenteis* was identified in COVID-19 patients compared to controls [11,25]. Although not statistically significant, the relative abundance of *Bifidobacterium adolescenteis* was shown to decrease with increasing disease severity [25]. In addition, *Bifidobacterium adolescenteis* was one of the most prevalent microbial taxa with the highest fold change in healthy individuals, and *Bifidobacterium longum* was found as a hub species in the control group by constructing a co-occurrence network at the species level [16]. *Bifidobacterium bifidum* was negatively correlated with the severity of COVID-19 [21] even after adjusting for antibiotic use and patient age.

The analysis using LEfSe revealed differences in *Bifidobacterium adolescentis* among samples of children. Comparing controls, the MIS-C group and the SARS-CoV-2 group, *Bifidobacterium adolescentis* was one of the most abundant species in the SARS-CoV-2 group, and an increase relative abundance of *Bifidobacterium adolescentis* also was observed in SARS-CoV-2 group [23].

On the other hand, in intestinal samples from COVID-19 patients compared to healthy controls, Rafiqul Islam et al. (2022) demonstrated that the genus *Bifidobacterium* was predominantly abundant [18]. In this way, at the species level, Li et al. (2021) reported an increase of *Bifidobacterium longum* in stool samples from COVID-19 patients, and the species *Bifidobacterium animalis*, was positively correlated with the severity of COVID-19 [14]. It was also demonstrated that the species *Bifidobacterium longum* showed a mean abundance increase of 30% in severe patients compared to mild patients, representing one of the four most abundant species associated with severe disease [22].

Post-COVID-19 patients were also evaluated. Relative abundance of the *Bifidobacterium* genus was significantly reduced in post-COVID-19 patients when compared to controls [10]. The differential relative abundance was also evaluated to demonstrate the specific effect of antibiotic therapy on post-COVID-19 patients. The results demonstrated a significant reduction of *Bifidobacterium* in post-COVID-19 patients who received antibiotic therapy during the acute phase of the disease compared to post-COVID-19 patients who did not receive the treatment [10].

In a study with post-acute COVID-19 syndrome (PACS) patients, the *Bifidobacterium pseudocatenulatum* species demonstrated higher inverse correlations at six months [15]. In addition, the comparison of the microbiota profile between patients with PACS and without PACS demonstrated basal composition gut bacteria was characterized by *Bifidobacterium* in patients without PACS (LEfSe), and the species *Bifidobacterium longum* at admission negatively correlated with PACS at 6 months.

In post-COVID-19 patients in the process of recovery, Cui et al. observed that the genus gradually increased. Furthermore, in correlation analysis between the intestinal microbiome and metabolomics in the gradual recovery process, a positive correlation between sphingosine-1-phosphate (S1P) and the *Bifidobacterium* genus was observed [9]. In another study, Yeoh et al. (2021) identified by LEfSe analysis *Bifidobacterium dentium* enrichment and *Bifidobacterium longum* depletion in gut microbiota samples from recovered patients [25].

In summary, the available evidence suggests that *Bifidobacterium* is associated with both protective effects and reduced abundance in relation to the disease. The genus has been found to be abundant in some cases and linked to disease severity. Studies have examined various stages of disease severity, comparing patients to controls as well as different severities and fatalities. Additionally, the recovery process and disease sequelae in post-COVID-19 patients, including those with post-acute COVID-19 syndrome (PACS), have been evaluated, with Bifidobacterium also being associated with these conditions.

### 4.3. The Probiotic Bifidobacterium in the Management of COVID-19

Bozkurt and Bilen evaluated the administration of only one strain, *Bifidobacterium* BB-12, in 20 cohort patients, but with additional treatment with antibiotics in the probiotic and non-probiotics group, and specific treatment (anti-Interleukin-6, anti-Interleukin-1, and immune plasma) in the non-probiotic group [26]. Significant differences were observed throughout the treatment in terms of hospitalization days, radiological improvement on the sixth day, and 3 weeks of hospitalization. In the probiotic-treated group, 19 out of 20 patients (95%) were discharged with an average hospital stay of 7.6 days, while 19 out of 24 (79%) patients without probiotic treatment were discharged with a median hospital stay of 13.6 days. The mortality rate was lower (5%) for the treated groups compared to the non-probiotic-treated group (20.8%). Findings in thorax CT showed differences between the groups at 6 days (3 days of treatment) and 3 weeks. While the early response rate was 13.6% in the non-probiotic group, the rate increased to 70.0% in the probiotic group. Similarly, at 3 weeks, the response rate was 28.6% in the non-probiotic group, while in the probiotic group, the rate increased to 100%. Plasma levels of IL-6 were also significantly decreased in the probiotic-treated group in the third week.

When comparing a group with active COVID-19 treated with a three-strain probiotic (*Bifidobacterium lactis*, *Lactobacillus salivarius*, and *Lactobacillus acidophilus*) and an untreated group, the results were more promising [32]. The mean values of fecal calprotectin were lower on days 3–5 in the groups administered probiotics compared to the control group. The lowest mean value was also observed between days 7–10 for the probiotic treatment group, where the mean value of fecal calprotectin was 124.9 ± 46 mg/dL, compared to 339.0 ± 102 mg/dL in the group without probiotic administration. The reduction in the level of the inflammatory marker from days 3–5 to days 7–10 was 35% for the probiotic group and only 16% for the non-probiotic group. During recruitment, the level of C-reactive protein (CRP) was similar between the groups. The decrease in the level of CRP was observed on days 3–5 and 7–10 for the probiotic-treated group compared to the untreated group. Regarding symptoms, the treated group showed a faster and continuous reduction in the need for oxygen use, while the untreated group had a lesser reduction. Additionally, four patients (10%) in the untreated group required 60% oxygen support, and three patients (7.5%) were admitted to the intensive care unit for at least 24 h. Meanwhile, no patient in the probiotic-treated group required 60% or higher levels of oxygen support. The average length of hospital stay was also shorter for the probiotic treatment group, at 14 ± 6 days compared to 19.0 ± 10 days for the untreated group.

Two studies evaluated the use of the same Sivomixx^®^ probiotic mix, containing *Streptococcus thermophilus*, *Lacticaseibacillus acidophilus*, *Lacticaseibacillus helveticus*, *Lacticaseibacillus paracasei*, *Lacticaseibacillus plantarum*, *Lacticaseibacillus brevis*, and *Bifidobacterium lactis* (DSM 32246 and DSM 32247) [27,28]. Both groups of patients had similar mean ages, but the observations varied between the disappearance of symptoms and mortality.

d’Ettorre et al. have been shown to be associated with the disappearance of diarrhea in all patients in 7 days [28]. A large proportion treated with probiotics (42.9%) resolved diarrhea within 24 h, and almost the totality (92.9%) within 3 days. From the second day of therapy, other symptoms as fever, asthenia, headache, myalgia, and dyspnea also showed a reduction. After seven days of treatment, patients treated with probiotics had an 8-fold significantly reduced risk of developing respiratory failure requiring resuscitation support (prone ventilation or extracorporeal membrane oxygenation) compared to those patients who did not receive probiotics.

Ceccarelli et al. conducted a retrospective observational cohort study that evaluated aspects related to the severity of COVID-19 cases [27]. The study compared patients with severe COVID-19 pneumonia who received the best available therapy (BAT- single drug or combination of the 2, 3, or 4 drugs) vs. patients treated with BAT and supplemented with oral bacteriotherapy in terms the rate of crude mortality, need for intensive care unit (ICU) admission, and length of hospitalization. A smaller number of patients who received bacteriotherapy died (10) vs. 34 who did not receive probiotics. The significant reduction in the risk of death for patients treated with BAT and oral bacteriotherapy was reconfirmed after adjustment. There were no differences in the need for ICU admission between groups, and the group treated with bacteriotherapy had a longer ICU stay compared to the group receiving BAT alone (20 days versus 14 days, respectively).

The administration of a probiotic mix containing three species of *Bifidobacterium* (*Bifidobacterium bifidum*, *Bifidobacterium longum* subsp. *infantis*, and *Bifidobacterium longum* subsp. *longum*) and *Lacticaseibacillus rhamnosus* showed a smaller effect at disease course [29]. In a prospective study, there was an average reduction of two days in the duration of viral diarrhea and prevention of hospital-acquired diarrhea for patients receiving a single antibiotic. However, there were no significant differences in the duration of illness, length of hospital stays, incidence of intensive care unit admission, oxygen support, or need for mechanical ventilation. There were also no differences in serum levels of biomarkers of systemic inflammation, renal function, and liver function.

Also, a retrospective study of 311 severe patients evaluated the potential effects of using probiotics on immunity and inflammation [31]. The study evaluated the use of the probiotic mix containing *Bifidobacterium infantis*, *Lactobacillus acidophilus*, *Dung enterococcus*, *Bacillus cereus*, *Bifidobacterium longum*, *Lactobacillus bulgaricus*, *Streptococcus thermophilus*, *Bacillus subtilis*. All the patients also received additional medicines, traditional Chinese medicine decoctions, and others. Specifically, the use of lopinavir/ritonavir, ribavirin b, and inhaled IFN-α was significantly higher in patients treated with probiotics and demonstrated higher multiple antibiotic ratios and longer antibiotic durations. Laboratory results indicated that the use of probiotics decreased CRP values in the group but did not differ from the values at T3 (last test before hospital discharge) compared to the non-probiotic group. The IL-6 levels increased over time in the probiotic group. However, total T lymphocytes, NK cells and B lymphocytes were upregulated in probiotic-treated patients but did not differ from the values at T3 compared with non-probiotic group. The CD4+/CD8+ ratio in probiotic-treated patients remained within the normal range, whereas without probiotics patients increased beyond the normal range.

The use of probiotics has also been evaluated in post-COVID-19 patients [30]. The mixed probiotic contained Lactobacilli (*Lactobacillus paracasei*, *Lactobacillus plantarum*, *Lactobacillus acidophilus*, and *Lactobacillus helveticus*), Bifidobacteria (*Bifidobacterium animalis* subsp. *lactis* BL03, previously identified *as B. longum*; *Bifidobacterium animalis* subsp. *lactis* BI04, previously identified as *B. infantis*; and *Bifidobacterium breve*), and *Streptococcus thermophilus*. The administration of a probiotic mix to 19 patients discharged after hospitalization for COVID-19 indicated that serum citrulline levels, IL-6, IL-12RA, and TNF-α were significantly reduced at the end of treatment (week 8) compared to baseline levels. However, zonulin and PV-1 showed no significant variation after treatment. After supplementation, there were changes in the gut microbiota profiling at the genus level, with *Ruminococcus*, *Oscillospira*, *Streptococcus*, and *Bifidobacterium* being more abundant, and statistically significant for the *Bifidobacterium* genus. The investigation of the gut microbiome in post-COVID-19 patients by machine learning after the administration of probiotics demonstrated that the important features were related to *Bifidobacterium*-related taxa. Additionally, a significant negative correlation was observed between citrulline and the genus *Bifidobacterium*.

Altogether, these studies suggest that *Bifidobacterium* probiotics may have beneficial effects in the management of COVID-19, including reducing hospitalization days, improving radiological outcomes, reducing inflammation markers, and enhancing immune response.

### 4.4. Risk of Bias and Quality Accessment

The risk of bias analysis identified that the biggest limitation of cross-sectional and case-control studies was the failure to identify confounding factors. Failure to identify and account for confounding factors can lead to biased results and inaccurate conclusions. In cross-sectional and case-control studies, the risk of confounding is particularly high due to their observational nature and the absence of temporal information. Therefore, researchers must carefully consider potential confounding variables and employ appropriate statistical methods to minimize their influence on study findings.

The summary of the microbiome methods used to identify and quantify the *Bifidobacterium* genus may also provide some hints on the quality of the papers. Several studies used the metagenomic shotgun for sequencing, which provides a detailed taxonomic analysis of all taxa observed in samples. In addition, most studies focused on amplicon sequencing used the V3–V4 region, which is the standard in microbiome studies. However, concerns arise when the databases and the statistical methods are considered. First, the great variety of databases and statistical methods make it difficult to make direct comparisons among studies because these variables can significantly influence the identification of a particular taxa or the quantification of its abundance. Second, several studies used non-conventional approaches, which may not be suitable or, or even inadequate in some cases. For example, the Greengenes is a very old and outdated database (last updated in 2013), while the NCBI database is very broad and doesn’t provide adequate alignment for taxonomy inference. Regarding the statistical analysis, while several studies used suitable methods for microbiome (e.g., LefSe or MaAsLin), other studies used simple statistics (e.g., Krustal-Wallis) or unusual methods (e.g., PELORA). In addition, two studies did not inform the database while two additional studies did not provide the specific statistical method used to assess the differential abundance of the genus *Bifidobacterium*, which is quite concerning.

Variations in the choice of primers, databases for taxonomy assignment, and statistical analysis used to identify differentially abundant taxa pose challenges in fully understanding the relationship between the *Bifidobacterium* genus and the COVID-19 disease since the equivalence of results can be impaired. Therefore, the adoption of reporting guidelines in human microbiome studies is highly recommended. One such guideline, known as the “Strengthening The Organization and Reporting of Microbiome Studies” (STORMS) checklist, has been proposed to enhance the quality of reporting in future human microbiome studies [40].

### 4.5. Evidence in Other Viral Infections

Given the significant impact of the COVID-19 pandemic on global health, there is a pressing need to understand how the immune system responds to the virus and identify potential therapeutic strategies to prevent and manage the disease. Recent studies have highlighted the crucial role of the gut microbiome in modulating the immune response and influencing susceptibility to respiratory infections. In this subsection, we will analyze the relationship of the genus *Bifibobacterium* with viral infections, in works that evaluated the composition and intervention.

In patients with H7N9 infection, the genus was drastically reduced in the intestinal microbiota compared with healthy control [41]. The evaluation of the intestinal microbiota of women infected with HIV, demonstrated by the analysis of LEfSe lower abundances of *Bifidobacterium* [42]. In an animal model, control chickens were differentially enriched with *Bifidobacterium* compared to chickens infected with influenza A subtype H9N2 virus [43].

In mice, *Bifidobacterium pseudolongum* and *Bifidobacterium animalis* levels are significantly elevated in surviving mice when compared to dead or mock-infected mice in H7N9 infection [44]. Also, the oral administration of the *Bifidobacterium animalis* or the combination with *Bifidobacterium pseudolongum* significantly reduces the severity of H7N9 infection in both antibiotic-treated and germ-free mice, suggesting a protective effect against influenza infections. Functional metagenomic analysis suggests that the anti-influenza effect mediated by *Bifidobacterium animalis* occurs via several specific metabolic molecules.

The use of *Bifidobacterium* as a probiotic has been evaluated as a treatment strategy for different viral infections. The first evidence of probiotic potential was demonstrated against respiratory tract infections in mice, where oral administration of *Bifidobacterium breve* protected against influenza infection [45].

The intranasal administration of *Bifidobacterium longum* with two different strains, reduces viral load, leading to a reduction in lung injury, and is strongly related to the survival of mice infected with the influenza virus [46]. Probiotic administration was associated with alterations in cellular recruitment to the lungs, reduction in the level of inflammatory cytokines and chemokines (IL-6, IP-10, and MCP-1), and an increase in protective cytokines (as interferon-λ and surfactant protein D).

The use of *Bifidobacterium longum* MM-2 in a murine model for the influenza virus demonstrated a protective effect by modulating the intestinal immune system in a similar way [47]. Oral administration of MM-2 has also been shown to reduce mortality, reduce inflammation in the lower respiratory tract, and decrease virus titers, in addition to reducing cell death and cytokines such as IL-6 and TNF-α in bronchoalveolar lavage. Changes were also observed in cellular activities in the lungs and spleen, through significant increases in NK cell activities and a significant increase in pulmonary gene expression of NK cell activators (IFN-γ, IL-2, IL-12, and IL-18). In uninfected mice, administration of MM-2 has also been shown to significantly increase IFN-γ production by Peyer’s patch cells and splenic NK cell activity.

The administration of *Bifidobacterium lactis* in children with gastroenteritis with rotavirus significantly reduced the duration of diarrhea, when compared to the control group and the group with the administration of another probiotic (*Saccharomyces boulardii*) [48]. It also represented the smallest group of patients who required hospitalization for intravenous hydration therapy.

Supplementation of healthy children between 3 and 5 years of age who did not receive any influenza vaccinations, was shown to reduce symptoms and the need for antibiotic treatment [49]. The probiotic group received *Lactobacillus acidophilus* NCFM or a combination *of Lactobacillus acidophilus* NCFM and *Bifidobacterium animalis* subsp *lactis* Bi-07. The combination of *Lactobacillus acidophilus* NCFM and *Bifidobacterium animalis* subsp *lactis* Bi-07 shows better results compared with the group received *Lactobacillus acidophilus* NCFM only. The combination group shows significantly lower odds of having rhinorrhea, cough, fever, or any of these symptoms relative to the placebo group.

Supplementation of healthy adults with minerals, vitamins, and probiotics from *Lactobacillus gasseri*, *Bifidobacterium longum,* and *Bifidobacterium bifidum* demonstrated that the mean duration of common cold episodes was significantly shorter in the probiotic-treated group when compared to the control group, and reduced gravity [50].

The use of the probiotic containing *Bifidobacterium breve* and *Limosilactobacillus mucosae* and mix improved the clinical symptoms of respiratory infection in a murine model [51]. The use of *Bifidobacterium breve* did not prevent weight loss in mice infected with influenza A, but the probiotic mix showed good results, better than the administration of *Limosilactobacillus mucosae* only. The use of *Bifidobacterium breve* has only been shown to significantly regulate inflammatory responses, which was not observed with *Limosilactobacillus mucosae* and the mix. However, administration of the mix demonstrated that the antiviral effects were associated with a significant decrease in the relative expression of the viral load and an increase in the expression of the antiviral protein MxA. The administration of the mix was also able to significantly increase the concentration of buritate, positively related to MxA expression, which may contribute to the relief of the clinical symptoms of the infection.

The use of different species and strains of *Bifidobacterium*, alone or in a mix of probiotics with other species, has been studied and evaluated as a treatment and prevention of viral infections. Its oral or intranasal administration demonstrates the ability to stimulate the innate immune system, controlling viral replication, reducing symptoms and lung damage, and is related to the survival of infected murine models.

### 4.6. Mechanisms of Protection of Bifidobacterium in COVID-19

There is growing evidence suggesting that gut microbiota, including the *Bifidobacterium* genus, plays a critical role in modulating the immune system and influencing the pathogenesis of viral infections, including COVID-19. In this subsection, we explore the potential mechanisms of action underlying the protective effects of *Bifidobacterium*, drawing on current knowledge of the interaction among the gut microbiota, the immune system, and viral infections. The protective function is related to its ability to modulate the host’s immune response. In the following paragraphs, we explore each mechanism in detail.

#### 4.6.1. Modulation of the Immune Response

The immune system is responsible for protecting the body against pathogens, infections, and foreign substances. *Bifidobacterium* is known to stimulate the immune system, and its administration has been shown to promote the production of proinflammatory cytokines such as interferon-gamma (IFN-γ), tumor necrosis factor-alpha (TNF-α), interleukin-2 (IL-2), interleukin-12 (IL-12), and interleukin-18 (IL-18), before viral infection in murine models, suggesting a protective effect against viral infections. This could be responsible for avoiding the imbalanced host response after SARS-CoV-2 infection to control the virus. In addition, an increase in natural killer cell (NK) activity, both in the spleen and in the lungs, was associated with reduced viral replication in murine models [47,52].

Higher titers of antibodies were also observed in mice treated with *Bifidobacterium*, demonstrating its ability to stimulate the humoral immune system response [52]. In this way, short-chain fatty acids (SCFA) produced by bacteria could inhibit histone deacetylase enzymes (HDAS), abolishing the differentiation of monocytes to dendritic cells and increasing the antimicrobial activity of macrophages [53].

Furthermore, studies with COVID-19 demonstrate that low levels of S1P meant a worse prognosis [9]. Low S1P levels predict ICU admission and hospital mortality, being suggested as S1P suggests as a novel biomarker of COVID-19 severity and morbidity [54]. In our review, we found a study that demonstrated that the increase in S1P protein was positively correlated with *Bifidobacterium* in recovered patients [9]. Furthermore, Kim et al. demonstrated that the greater abundance of the genus *Bifidobacterium* in the salivary microbiome was related to lower levels of IL-17F and MCP-1. These cytokines play a crucial role in regulating the immune response to viral infections and may help prevent the cytokine storm that is associated with severe COVID-19.

#### 4.6.2. Reduction of Inflammation

Inflammation is a hallmark of COVID-19, and excessive inflammation can cause significant damage to the lungs and other organs. *Bifidobacterium* has been shown to reduce inflammation by regulating the production of pro-inflammatory cytokines and decreasing levels of IL-6 and TNF-α [47,52,55]. Likewise, SCFA has been linked to an anti-inflammatory role in neutrophils and dendritic cells, increasing anti-inflammatory mediators and reducing inflammatory cytokines [51]. This anti-inflammatory effect may help prevent the development of severe COVID-19.

#### 4.6.3. Competitive Advantages with Pathogenic Microbes

The competition between species is important for the formation of the gut communities and the maintenance of the host’s healthy microbiota. The gut microbiota plays a critical role in maintaining a healthy gut environment and preventing the overgrowth of pathogenic microbes. *Bifidobacterium* has been shown to compete with pathogenic bacteria such as *Escherichia coli* and *Salmonella typhimurium* by antibacterial substances secreted or competition for common adhesive sites in the gut [56,57,58,59]. In this way, the competitive ability demonstrated by Bifidobacterium can avoid the installation of other pathogens and may help prevent gut dysbiosis, which has been associated with COVID-19 severity.

#### 4.6.4. Maintenance of Gut Barrier Function

The gut barrier is a functional unit and a critical component of the immune system, forming a physical and functional barrier that protects the host. The disruption has been associated with the development of systemic inflammation and autoimmune diseases. *Bifidobacterium* has been shown to reduce gut permeability [60] and to promote the production of mucins and expression of tight junction proteins in intestinal cells, which are critical for maintaining the gut barrier, role also attributed to SCFA [53,61]. This can be critical to help prevent the translocation of pathogenic bacteria and endotoxins into the bloodstream, a phenomenon that has been associated with severe COVID-19.

## 5. Conclusions

In conclusion, the observational studies indicate that *Bifidobacterium* was associated with both protective effects and reduced abundance in relation to the disease. The genus has been found to be abundant in some cases and linked to disease severity. The studies evaluating the use of *Bifidobacterium* as probiotics have demonstrated the potential of this genus in reducing symptoms, improving pulmonary function, reducing inflammatory markers, alleviating gastrointestinal symptoms, and even contributing to better control of mortality. As potential mechanisms of action, *Bifidobacterium* may offer protection against COVID-19 through its ability to modulate the immune response, reduce inflammation, compete with pathogenic microbes, and maintain gut barrier function. Through this analysis, we provide insights into the potential of *Bifidobacterium* as a therapeutic agent in the context of COVID-19 and shed light on possible avenues for future research.

## Figures and Tables

**Table 1:** Paper details identified from the systematic search with results for the genus *Bifodobacterium*.

Study	Country	Type of Study	Type of Sample	NGS Tecnology	Type of Sequencing	N	Groups	Abundance in COVID-19 Group	Clinical Relevance
Albrich et al., 2022 [8]	Switzerland and Ireland	Cohort	Stool	MiSeq	V3–V4	128 ^1^	32 mild/ moderate 45 severe/non-fatal 22 severe/fatal COVID-19 29 healthy controls	Decreased	Associated with severity
Cui et al., 2022 [9]	China	Case-control	Tongue-coating and stool	MiSeq	V3–V5	195	35 patients post-COVID-19 160 healthy controls	No statistical difference observed	Not associated with recovery process
Ferreira-Junior et al., 2022 [10]	Brazil	Case-control	Stool	MiSeq	V3–V4	220	149 post-COVID-19 71 healthy controls	Decreased in post-COVID-19 and antibiotic-treated	Associated with disease and antibiotic treatment
Gumenyuk et al., 2022 [11]	Russia	Cross-sectional	Stool	SOLiD 5500	Shotgun	208	110 COVID-19 patients 98 healthy controls	Decrease of *B. adolescentis*	Associated with disease
Hazan et al., 2022 [12]	USA	Cross-sectional	Stool	NextSeq 500/550	Shotgun	70	50 COVID-19 patients 20 exposed controls	Decreased	Associated with severity
Kim et al., 2023 [13]	USA	Cohort	Saliva and nasopharyngeal	MiSeq	V1–V2	144	114 samples COVID-19 positive 30 samples COVID-19 negative	Decreased in ICU group. The genus was associated with lower levels of IL-17F and MCP-1	Associated with severity
Li et al., 2021 [14]	China	Case-control	Stool	BGISEQ-500	Shotgun	66	47 COVID-19 patients 19 healthy controls	Increase of *B. longum* in COVID-19. *B. bifidum* was negatively correlated with the severity and *B. animalis* was positively correlated.	Associated with severity
Liu et al., 2022 [15]	China	Cohort	Stool	NextSeq 550	Shotgun	174	106 COVID-19 (50 PACS) 68 non-COVID-19	Decreased. *B. pseudocatenulatum* was inversely correlated with PACS at 6 months	Associated with severity and PACS
Maddah et al., 2023 [16]	China ^2^/Iran ^3^	Cross-sectional	Stool	MiSeq	V3–V4	30	30 COVID-19 patients 30 healthy controls	Decrease of *B. adolescentis*	Associated with disease
Mazzarelli et al., 2022 [17]	Italy	Cohort	Stool	MiSeq	V3–V4	97	47 mild COVID-19 50 severe COVID-19	No statistical difference observed	Not associated with severity
Rafiqul Islam et al., 2022 [18]	Bangladesh	Cross-secctional	Stool and saliva	MiSeq	V3–V4	37	22 COVID-19 patients 15 healthy controls	Decreased	Associated with disease
Reinold et al., 2021 [19]	Germany	Cross-secctional	Stool	Novaseq 6000	V3–V4	212	44 mild COVID-19 35 moderate COVID-19 38 severe/critical COVID-19 95 SARS-CoV-2 negative controls	Decreased	Associated with disease
Romani et al., 2022 [20]	Italy	Cohort	Stool	MiSeq	V3–V4	183	68 COVID-19 children 16 non-COVID-19 4 children with MIS	Decreased	Associated with disease
Rueca et al., 2021 [21]	Italy	Cohort	Nasal/ oropharyngeal	IonS5	V2-4-8 e V3-6 e 7-8	39	21 COVID-19 patients 8 HCoV patients 10 healthy controls	Complete depletion in ICU patients	Associated with severity
Sun et al., 2022 [22]	China	Case-control	Stool	Novaseq 6000	Shotgun	71	63 COVID-19 patients 8 non-infected controls	Increase of *B. longum* and depletion of *B. pseudocatenulatum* in COVID-19	Associated with severity
Suskun et al., 2022 [23]	Turkey	Cohort	Stool	NovaSeq 6000	V3–V4	39	20 COVID-19 children 25 MIS-C children 19 healthy controls	Increase of *B. adolescentis* in COVID-19	Associated with severity
Wang et al., 2023 [24]	China	Cohort	Stool	MiSeq	V3–V4	186	59 COVID-19 children 50 asymptomatic caregivers 52 healthy children 25 healthy adults	Decreased	Associated with disease
Yeoh et al., 2021 [25]	China	Case-control	Stool	NovaSeq 6000	Shotgun	178	100 COVID-19 patients 78 non-COVID-19 patients	Depletion of *B. adolescentis* in COVID-19. *B. bifidum* was negatively correlated with severity. Increase of *B. dentium* and depleted *B. longum* in recovered patients.	Associated with disease severity and recovery process

^1^ Patients sequenced. ^2^ Original data. ^3^ Data reanalysis. MIS: multisystem inflammatory syndrome.

**Table 2:** Paper details identified from the systematic search with results for the genus *Bifodobacterium* as probiotics strains.

Study	Country	N	Groups	Probiotics	Outcome	Clinical Relevance
Bozkurt & Bilen, 2021 [26]	Turkey	44	probiotic group (20) non-probiotic group (24)	*Bifidobacterium* BB-12	Reduction of hospitalization days; thorax resolution at 6 days and 3 weeks; reduction in IL-6 plasma levels.	Beneficial
Ceccarelli et al., 2020 [27]	Italy	200	probiotic group (88) non-probiotic group (112)	Sivomixx^®^ containing *Streptococcus thermophilus* DSM 32245, *Bifidobacterium lactis* DSM 32246, *Bifidobacterium lactis* DSM 32247, *Lactobacillus acidophilus* DSM 32241, *Lactobacillus helveticus* DSM 32242, *Lactobacillus paracasei* DSM 32243, *Lactobacillus plantarum* DSM 32244, and *Lactobacillus brevis* DSM 27961	Significant reduction in the risk of death.	Beneficial
d’Ettorre et al., 2020 [28]	Italy	70	probiotic group (28) non-probiotic group (42)	Sivomixx^®^ containing *Streptococcus thermophilus* DSM 32345, *Lactobacillus acidophilus* DSM 32241, *Lactobacillus helveticus* DSM 32242 *Lactobacillus paracasei* DSM 32243, *Lactobacillus plantarum* DSM 32244, *Lactobacillus brevis* DSM 27961, *Bifidobacterium lactis* DSM 32246, and *Bifidobacterium lactis* DSM 32247	Remission of diarrhea in almost all patients treated within 72 h, reduction in other symptoms, 8x lower risk of developing respiratory failure.	Beneficial
Ivashkin et al., 2021 [29]	Russian	202	probiotic group (101) non-probiotic group (101)	Florasan-D containing *Lacticaseibacillus rhamnosus* PDV 1705, *Bifidobacterium bifidum* PDV 0903, *Bifidobacterium longum* subsp. *infantis* PDV 1911, and *Bifidobacterium longum* subsp. *longum* PDV 2301	Average reduction of two days in the duration of viral diarrhea and prevention of hospital-acquired diarrhea for patients receiving a single antibiotic.	Beneficial
Laterza et al., 2023 [30]	Italy	19	post-COVID-19 patients (19)	VSL#3^®^ (lot number 909031) containing *Lactobacillus paracasei* BP07, *Lactobacillus plantarum* BP06, *Lactobacillus acidophilus* BA05, *Lactobacillus helveticus* BD08 (previously identified as *L. delbrueckii* subsp. *bulgaricus), Bifidobacterium animalis* subsp. *lactis* BL03 (previously identified as *B. longum*), *Bifidobacterium animalis* subsp. *lactis* BI04 (previously identified as *B. infantis*), *Bifidobacterium breve* BB02, and *Streptococcus thermophilus* BT01	Significant reduction of IL-6, TNF-ALFA, IL-12RA, and citrulline.	Beneficial
Li et al., 2021 [31]	China	311	probiotic group (123) non-probiotic group (188)	*Bifidobacterium infantis, Lactobacillus acidophilus, Dung enterococcus, Bacillus cereus, Bifidobacterium longum, Lactobacillus bulgaricus, Streptococcus termófilos, Bacillus subtilis, Enterococcus faecium,* and *Bacillus subtilis*	No significant differences were observed.	No difference
Saviano et al., 2022 [32]	Italy	80	probiotic group (40) non-probiotic group (40)	Lactibiane Iki^®^ containing *Bifidobacterium lactis* LA 304, *Lactobacillus salivarius* LA 302, and *Lactobacillus acidophilus* LA 201	Lower values of fecal calprotectin, reduction of the inflammatory marker CRP, faster and continuous reduction needed for O_2_ support, and lower mean length of hospitalization.	Beneficial

## Data Availability

Not applicable.

## Supplementary Materials

The following supporting information can be downloaded at https://www.mdpi.com/article/10.3390/life13091847/s1, File S1: Search strategy for each database.
