# Supplementary material for: The Role of Bifidobacterium in COVID-19: A Systematic Review
Source: Life (Basel). 2023 Aug 31;13(9):1847. doi: 10.3390/life13091847 (PMC10532519; doi:10.3390/life13091847)
Supplement: Supplementary file 1 [file life-13-01847-s001.zip › life-2533468-supplementary.pdf]

Supplementary File 1. Search strategy for each database.

Search strategy to PubMed/MEDLINE:

(COVID-19[mh] OR COVID19[tw] OR 2019 Novel Coronavirus Disease[tw] OR 2019 Novel Coronavirus Infection[tw] OR COVID-19 Pandemic*[tw] OR COVID-19 Virus Disease[tw] OR COVID-19 Virus Infection[tw] OR Coronavirus Disease 2019[tw] OR Coronavirus Disease-19[tw] OR SARS Coronavirus 2 Infection[tw] OR SARS-CoV-2 Infection[tw] OR SARS Coronavirus 2[tw] OR SARS-CoV-2 Virus[tw] OR Severe Acute Respiratory Syndrome Coronavirus 2 Infection[tw] OR SARS-CoV-2[mh] OR 2019 Novel Coronavirus[tw] OR COVID-19 Virus[tw] OR COVID19 Virus[tw] OR Coronavirus Disease 2019 Virus[tw] OR Severe Acute Respiratory Syndrome Coronavirus 2[tw] OR Wuhan Coronavirus[tw] OR coronavirus[tw]) AND (Bifidobacterium[mh] OR Bifidobacterium[tw])

Filter: Language: English; Years: 2020-2023

Search strategy to EMBASE:

('coronavirus disease 2019'/exp OR 'coronavirus disease 2019':ti,ab,kw OR ‘2019 Novel Coronavirus Disease’:ti,ab,kw OR ‘2019 Novel Coronavirus Infection’:ti,ab,kw OR ‘COVID-19 Pandemic*’:ti,ab,kw OR ‘COVID-19 Virus Disease’:ti,ab,kw OR ‘COVID-19 Virus Infection’:ti,ab,kw OR ‘Coronavirus Disease 2019’:ti,ab,kw OR ‘Coronavirus Disease-19’:ti,ab,kw OR ‘SARS Coronavirus 2 Infection’:ti,ab,kw OR ‘SARS-CoV-2 Infection’:ti,ab,kw OR ‘SARS Coronavirus 2’:ti,ab,kw OR ‘SARS-CoV-2 Virus’:ti,ab,kw OR ‘Severe Acute Respiratory Syndrome Coronavirus 2 Infection’:ti,ab,kw OR 'Severe acute respiratory syndrome coronavirus 2'/exp OR ‘2019 Novel Coronavirus’:ti,ab,kw OR ‘COVID-19 Virus’:ti,ab,kw OR ‘COVID19 Virus’:ti,ab,kw OR ‘Coronavirus Disease 2019 Virus’:ti,ab,kw OR ‘Severe Acute Respiratory Syndrome Coronavirus 2’:ti,ab,kw OR ‘Wuhan Coronavirus’:ti,ab,kw OR ‘coronavirus’:ti,ab,kw) AND (Bifidobacterium/exp OR Bifidobacterium:ti,ab,kw)

Filter: Language: English; Years: 2020-2023

Search strategy to Scopus:

TITLE-ABS(“coronavirus disease 2019” OR “2019 Novel Coronavirus Disease” OR “2019 Novel Coronavirus Infection” OR “COVID-19 Pandemic*” OR “COVID-19 Virus Disease” OR “COVID-19 Virus Infection” OR “Coronavirus Disease 2019” OR “Coronavirus Disease-19” OR ”SARS Coronavirus 2 Infection” OR “SARS-CoV-2 Infection” OR “SARS Coronavirus 2” OR “SARS-CoV-2 Virus” OR “Severe Acute Respiratory Syndrome Coronavirus 2 Infection” OR “Severe acute respiratory syndrome coronavirus 2” OR “2019 Novel Coronavirus” OR “COVID-19 Virus” OR “COVID19 Virus” OR “Coronavirus Disease 2019 Virus” OR “Severe Acute Respiratory Syndrome Coronavirus 2” OR “Wuhan Coronavirus” OR coronavirus) AND TITLE-ABS(Bifidobacterium)

Filter: Language: English; Years: 2020-2023

Search strategy to Web of Science:

(COVID-19 OR 2019 Novel Coronavirus Disease OR 2019 Novel Coronavirus Infection OR COVID-19 Pandemic* OR COVID-19 Virus Disease OR COVID-19 Virus Infection OR Coronavirus Disease 2019 OR Coronavirus Disease-19 OR SARS Coronavirus 2 Infection OR SARS-CoV-2 Infection OR SARS Coronavirus 2 OR SARS-CoV-2 Virus OR Severe Acute Respiratory Syndrome Coronavirus 2 Infection OR SARS-CoV-2 OR 2019 Novel Coronavirus OR COVID-19 Virus OR COVID19 Virus OR Coronavirus Disease 2019 Virus OR Severe Acute Respiratory Syndrome Coronavirus 2 OR Wuhan Coronavirus OR coronavirus) AND (Bifidobacterium)

Filter: Language: English; Years: 2020-2023
